# Phase separation, transcriptional elongation control, and human diseases

**DOI:** 10.1093/jmcb/mjab023

**Published:** 2021-04-05

**Authors:** Chenghao Guo, Zhuojuan Luo, Chengqi Lin

**Affiliations:** 1 School of Life Science and Technology, Key Laboratory of Developmental Genes and Human Disease, Southeast University, Nanjing 210096, China; 2 Co-innovation Center of Neuroregeneration, Nantong University, Nantong 226019, China

Precise regulation of gene transcription is of great importance to development and diseases. Promoter-proximal transcriptional pause is a key and general mechanism to precisely control transcription in metazoans. Subsequent to transcription initiation and synthesis of a short RNA, RNA polymerase II (Pol II) usually pauses at the promoter-proximal regions, standing by for further signals to be released into the productive elongation stage. Fine regulation of Pol II pausing and release is achieved by the concerted action of many negative and positive elongation factors, including the super elongation complex (SEC). Recent studies suggested that phase-separated assemblies of transcription regulatory complexes could provide a general biophysical basis for the dynamic regulation of transcription in response to various cellular needs, though direct evidence at endogenous level in living cells is still largely lacking. Here, we summarize and discuss latest advances in understanding how phase separation contributes to RNA polymerase II-mediated transcription, with a focus on transcriptional pause and release.

## Promoter-proximal pausing of RNA Pol II

RNA Pol II-mediated transcription begins with the assembly of the pre-initiation complex (PIC), which contains polymerase, general transcription factors (TFs), and a large number of coactivators. The final essential step in the assembly of PIC is the recruitment of transcription factor IIH (TFIIH), which not only initiates transcription by opening the double-stranded DNA but also phosphorylates Ser5 and Ser7 residues of Pol II C-terminal domain (CTD) through its catalytic subunit cyclin-dependent kinase 7 (CDK7) (Kwak and Lis, 2013). Ser5-phosphorylated Pol II CTD can recruit capping enzymes to modify the 5′ end of the nascent RNA as well as recruit negative transcription elongation factors to arrest Pol II in the proximal region of the promoter (Kwak and Lis, 2013).

The phenomenon of promoter-proximal Pol II pausing was first described in *Drosophila melanogaster*. A large amount of Pol II was detected between nucleotides −12 and +65 of the *HSP70* gene under the nonheat shock condition (Gilmour and Lis, 1986). Subsequently, promoter-proximally paused Pol II was also found in the proto-oncogenes *FOS* and *MYC* (Plet et al., 1995). With the maturation and wide application of genome-wide profiling approaches, more evidences have shown that promoter-proximal pausing of Pol II is a common event during gene transcription, particularly for the genes related to development and stress response (Kwak and Lis, 2013). Promoter-proximal Pol II pausing is the rate-limiting step and check point during early transcription elongation, at which transcription is halted until the proper assembly of elongation machinery.

The promoter-proximal pausing step is mainly mediated by the negative elongation factor (NELF) and 5,6-dichloro-1-β-D-ribofuranosylbenzimidazole (DRB) sensitivity-inducing factor (DSIF). NELF comprises four subunits: NELF-A, NELF-B, NELF-C/D, and NELF-E, while DSIF is composed of SPT4 and SPT5. Similar to Pol II CTD, the C-terminal region of SPT5 has a repeat region that can be phosphorylated by both TFIIH and positive transcription elongation factor b (P-TEFb). The release of paused Pol II into productive elongation is triggered by P-TEFb-containing complexes (Adelman and Lis, 2012). By interacting with TFs and cofactors, P-TEFb is directly or indirectly recruited to promoter region to phosphorylate Ser2 residue of Pol II CTD, NELF, and DSIF. Upon phosphorylation, NELF dissociates with Pol II, while DSIF promotes transcription elongation (Kwak and Lis, 2013).

## Regulation of transcriptional pause and release

*In vitro* transcriptional elongation assays showed that the DSIF and NELF complexes can arrest Pol II around the promoter. In the presence of DSIF, NELF can effectively inhibit transcription *in vitro* (Wada et al., 1998; Yamaguchi et al., 1999). Biochemical and structural biology studies have suggested that DSIF and NELF can directly interact with Pol II, upstream template DNA, and newly synthesized RNA, thereby helping in maintaining the pause stage of Pol II as well as inhibiting premature termination of transcription (Vos et al., 2018). A recent study has demonstrated that destruction of the NELF complex can cause Pol II to shift downstream from the pause site, resulting in early termination of transcription. This finding indicates the importance of NELF in maintaining Pol II pause at the correct position (Aoi et al., 2020). In addition, the transcription elongation factor ELL3, which plays a key role in the activation of the developmentally regulated gene during embryonic stem cell (ESC) differentiation, can function in establishing Pol II pause from the enhancers of these genes (Lin et al., 2013).

In response to developmental signals or environmental cues, paused Pol II can be released by P-TEFb, which is composed of CDK9 and its regulatory subunits CCNT1/2 (Ni et al., 2008). P-TEFb mainly exists in three different complexes: the SEC family members (Lin et al., 2010; Luo et al., 2012a), the bromodomain-containing protein 4 (BRD4)‒P-TEFb complex (Jang et al., 2005; Yang et al., 2005), and the inactive 7SK small nuclear ribonucleoprotein‒P-TEFb complex (Nguyen et al., 2001; Yang et al., 2001). P-TEFb shuttles between the inactive and active states based on cellular transcriptional demand. SEC, one of the most active P-TEFb-containing complexes, is essential for rapid gene activation in response to stress or developmental signals (Lin et al., 2011; Luo et al., 2012b). The multisubunit macromolecular protein complex SEC was originally discovered through identification of the complexes containing mixed lineage leukemia (MLL) fusion protein. Besides the transcription elongation factors P-TEFb and ELL family members, SEC also contains the AF4/FMR2 family member 1/4, ELL binding factor EAF1/2 (Lin et al., 2010), and YEATS domain protein family member ENL or AF9. It has been shown recently that SEC is assembled as the phase-separated droplets *in vivo*, and that its capability in phase separation is required for extracting and concentrating P-TEFb from the inactive complex (Guo et al., 2020).

## Phase separation in transcription

**Figure 1:**
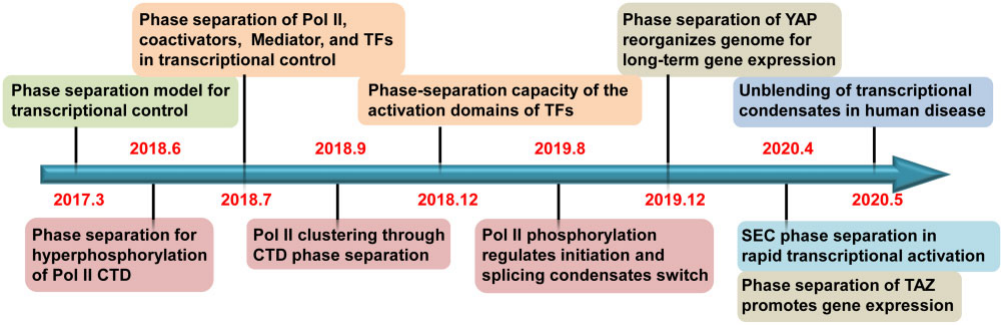
The milestones of phase separation in transcriptional regulation.

Numerous studies have shown that biological macromolecules, including proteins and RNAs, can self-assemble into nonmembrane cellular compartments via liquid–liquid phase separation (LLPS). These phase-separated biocondensates allow biomolecules to rapidly move inside and exchange with outside factors, enabling effective bioreactions (Shin and Brangwynne, 2017). Transcription is highly regulated to ensure proper temporal and spatial gene expression pattern. Transcription regulation involves concerted actions from various TFs and chromatin regulators, some of which have been recently demonstrated to function in phase-separated condensates (Figure 1). It has been reported that homologous mammalian proteins YAP and TAZ, two key TFs in the Hippo signaling pathway, are activated by LLPS to regulate gene transcription upon hyperosmotic stress (Franklin and Guan, 2020). Studies using live-cell high-resolution microscopy have also shown that Pol II and Mediator are dynamically concentrated in phase-separated droplets in a transcription inhibitor-sensitive manner to regulate genes controlled by super-enhancers (SEs) in murine ESCs (Cho et al., 2018; Sabari et al., 2018). Inhibition of transcription by small-molecule inhibitors leads to disruption of transcriptional condensates. For examples, JQ1, the selective inhibitor of the bromodomain and extraterminal domain protein family, can dissolve the condensates containing Pol II and Mediator, which co-condenses with BRD4 at SE clusters (Cho et al., 2018; Sabari et al., 2018). In contrast, DRB specifically inhibits stable Pol II condensates without affecting the Mediator clusters (Cho et al., 2018; Sabari et al., 2018).

Weak and multivalent interactions among proteins bearing low-complexity domains (LCDs) or intrinsically disordered regions (IDRs) are believed to be one of the main driving forces for phase separation (Shin and Brangwynne, 2017). Many eukaryotic TFs consist of fixed structured DNA-binding domains and LCDs or IDRs containing transactivation domains (ADs). Although it remains unclear whether phase separation is absolutely required for the function of LCD or AD-containing TFs in living cells at their endogenous levels (Chong et al., 2018), the ability of LCD or AD to form phase-separated droplets could be one of the key features of TFs and important for the interaction of TFs with the Mediator coactivator (Boija et al., 2018). In addition, Pol II forms condensates at active genes through its low-complexity CTD that undergoes cooperative LLPS to concentrate transcription regulators to initiate transcription (Kwon et al., 2013; Boehning et al., 2018; Lu et al., 2018). It will be interesting to see, as more definite experiments are carried out to address the functional role, if any, of phase separation in RNA Pol II, TFs, and coactivators forming at *cis*-regulatory elements that activate transcription under physiologically relevant conditions.

## Phase separation in transcriptional pause and release

**Figure 2:**
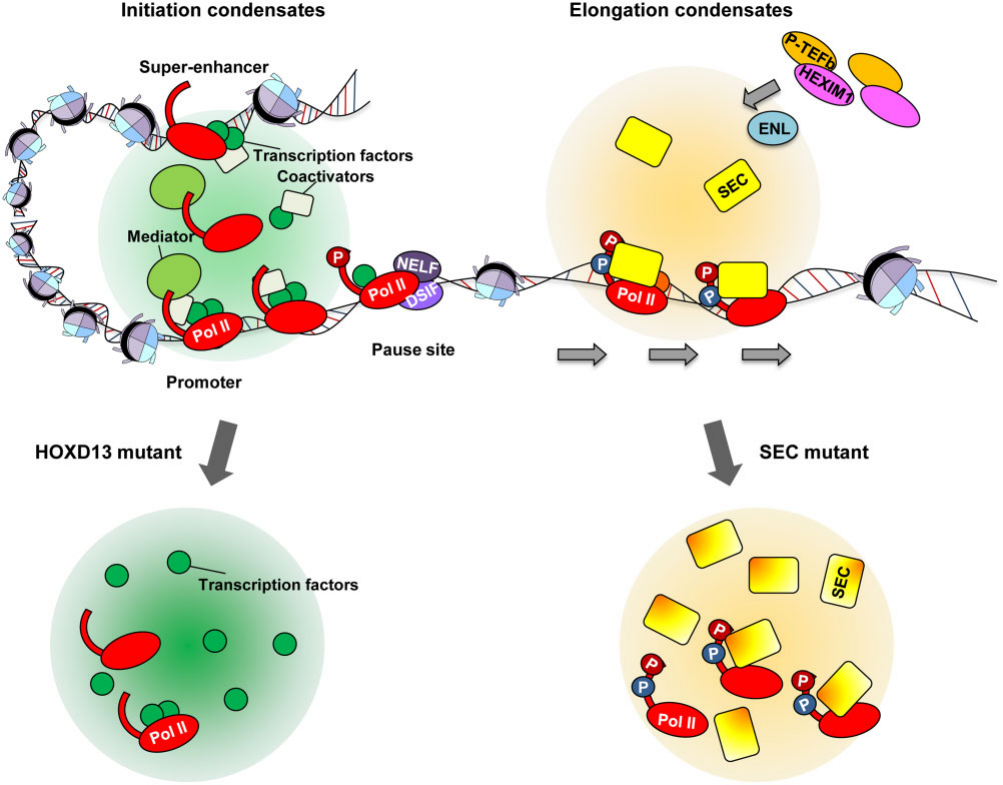
Phase separation in promoter-proximal pause and release. RNA Pol II and its regulatory factors can form dynamic phase-separated condensates within cells, regulating transcription. P-TEFb can be extracted from the inactive 7SK/HEXIM1 complex by the SEC core component ENL via LLPS, and thus is incorporated into the SEC droplets to activate transcription. Unblending of transcriptional condensates via the expansion of amino acid repeats in HOXD13 may cause the disease synpolydactyly. Mutations of SEC components, such as fusion with MLL in leukemia and mutations of the ENL YEATS in Wilms tumor, increase the capability of SEC to phase-separate.

Phase separation is also involved in the release of paused Pol II into productive elongation stage through dynamic exchange of related factors at the promoter-proximal regions. The histidine-rich domain of CCNT1, a subunit of P-TEFb, forms phase-separated condensates to enrich RNA Pol II, thereby leading to Pol II CTD hyperphosphorylation and enhancing transcriptional elongation activity (Lu et al., 2018). In addition, the transcription elongation complex SEC executes its rapid transcriptional activation function in the form of phase-separated droplets. Upon serum exposure, SEC can rapidly assemble into phase-separated droplets at the immediate early genes, triggering rapid transcription induction from pausing state (Guo et al., 2020; Figure 2). ENL and AFF4, as the scaffolds of SEC, can dynamically extract and concentrate the kinase module P-TEFb from the inactive HEXIM1‒P-TEFb complex by LLPS, so as to promote the rapid assembly of SEC (Guo et al., 2020; Figure 2). Disruption of SEC or SEC droplets by ENL depletion significantly reduces the efficiency of transcripts synthesis, resulting in the inability of cells to response to stress (Guo et al., 2020). It would be worthy further examining whether changes in SEC phase-separation capabilities or droplet properties will directly affect pause release.

Pol II CTD is intrinsically disordered and lowly complex. A recent study has shown that the switch from hypophosphorylated to hyperphosphorylated state of Pol II CTD drives the transition of Pol II from transcription initiation to RNA splicing condensates (Guo et al., 2019). RNA splicing occurs during transcription elongation. Promoter-proximal pausing of Pol II is the intermediate stage between transcription initiation and elongation. However, it remains unclear how paused Pol II is released into the elongation condensates. What are the differences of these stage-different Pol II condensates? These intriguing aspects regulating Pol II behaviors during transcription need to be further investigated. Single molecule-based imaging approaches could promote the research in this regard (Steurer et al., 2018).

## Mutations, abnormal phase separation, and diseases

Misregulation in promoter-proximal Pol II pause and release can lead to developmental abnormalities and human diseases, including cancers. A recent study has suggested that unblending of transcriptional condensates via the expansion of amino acid repeats in TFs may cause the disease synpolydactyly (Basu et al., 2020; Figure 2). Fusion of the SEC core component ENL to MLL can greatly augment the capability of SEC to phase-separate, which might contribute to misactivation of leukemic genes (Guo et al., 2020; Figure 2). In Wilms tumor, mutations in the ENL YEATS domain can promote the self-assembly of ENL via phase separation, resulting in the recruitment of SEC to promote oncogene expression and tumorigenesis (Wan et al., 2020; Figure 2). Thus, disease-related mutations in transcriptional regulators may be linked to phase separation models by changing their properties and functional activity.

## Concluding marks

Transcriptional regulation is a highly complex process. It is challenging to fully understand the regulatory processes of transcriptional pause, release, and productive elongation. The phase separation model helps in understanding how different factors, including Pol II, TFs, coactivators, chromatin regulators, and RNA-binding proteins, are recruited to maintain the homeostasis of gene expression, and how cells rapidly change their gene expression programs in response to signaling. However, we have to be aware that direct measurements are still lacking regarding the capabilities and properties of phase-separated condensates under endogenous conditions and also the causal relationship between LLPS and transcriptional regulation (McSwiggen et al., 2019). Deciphering the self-organization of transcriptional regulatory complexes via phase separation under physiological condition would help further understanding the complicated gene expression program as well as provide potential accurate targets for therapeutic strategies in related human diseases.

*[We are grateful to the members in Lin & Luo Lab for critical discussion. This work was financially supported by grants from the National Key R&D Program of China (2018YFA0800100 and 2018YFA0800101 to C.L.; 2018YFA0800103 to Z.L.) and the National Natural Science Foundation of China (32030017 and 31970626 to C.L.; 31970617 to Z.L.)]*

## References

[mjab023-B1] Adelman K. , Lis J.T. (2012). Promoter-proximal pausing of RNA polymerase II: emerging roles in metazoans. Nat. Rev. Genet. 13, 720–731. 22986266 10.1038/nrg3293 PMC3552498

[mjab023-B2] Aoi Y. , Smith E.R., Shah A.P., et al (2020). NELF regulates a promoter-proximal step distinct from RNA Pol II pause-release. Mol. Cell 78, 261–274.e5. 32155413 10.1016/j.molcel.2020.02.014 PMC7402197

[mjab023-B3] Basu S. , Mackowiak S.D., Niskanen H., et al (2020). Unblending of transcriptional condensates in human repeat expansion disease. Cell 181, 1062–1079.e30. 32386547 10.1016/j.cell.2020.04.018 PMC7261253

[mjab023-B4] Boehning M. , Dugast-Darzacq C., Rankovic M., et al (2018). RNA polymerase II clustering through carboxy-terminal domain phase separation. Nat. Struct. Mol. Biol. 25, 833–840. 30127355 10.1038/s41594-018-0112-y

[mjab023-B5] Boija A. , Klein I.A., Sabari B.R., et al (2018). Transcription factors activate genes through the phase-separation capacity of their activation domains. Cell 175, 1842–1855.e16. 30449618 10.1016/j.cell.2018.10.042 PMC6295254

[mjab023-B6] Cho W.K. , Spille J.H., Hecht M., et al (2018). Mediator and RNA polymerase II clusters associate in transcription-dependent condensates. Science 361, 412–415. 29930094 10.1126/science.aar4199 PMC6543815

[mjab023-B7] Chong S. , Dugast-Darzacq C., Liu Z., et al (2018). Imaging dynamic and selective low-complexity domain interactions that control gene transcription. Science 361, eaar2555. 29930090 10.1126/science.aar2555 PMC6961784

[mjab023-B8] Franklin J.M. , Guan K.L. (2020). YAP/TAZ phase separation for transcription. Nat. Cell Biol. 22, 357–358. 32203419 10.1038/s41556-020-0498-8

[mjab023-B9] Gilmour D.S. , Lis J.T. (1986). RNA polymerase II interacts with the promoter region of the noninduced hsp70 gene in Drosophila melanogaster cells. Mol. Cell. Biol. 6, 3984–3989. 3099167 10.1128/mcb.6.11.3984-3989.1986 PMC367162

[mjab023-B10] Guo C. , Che Z., Yue J., et al (2020). ENL initiates multivalent phase separation of the super elongation complex (SEC) in controlling rapid transcriptional activation. Sci. Adv. 6, eaay4858. 32270036 10.1126/sciadv.aay4858 PMC7112754

[mjab023-B11] Guo Y.E. , Manteiga J.C., Henninger J.E., et al (2019). Pol II phosphorylation regulates a switch between transcriptional and splicing condensates. Nature 572, 543–548. 31391587 10.1038/s41586-019-1464-0 PMC6706314

[mjab023-B12] Jang M.K. , Mochizuki K., Zhou M., et al (2005). The bromodomain protein Brd4 is a positive regulatory component of P-TEFb and stimulates RNA polymerase II-dependent transcription. Mol. Cell 19, 523–534. 16109376 10.1016/j.molcel.2005.06.027

[mjab023-B13] Kwak H. , Lis J.T. (2013). Control of transcriptional elongation. Annu. Rev. Genet. 47, 483–508. 24050178 10.1146/annurev-genet-110711-155440 PMC3974797

[mjab023-B14] Kwon I. , Kato M., Xiang S., et al (2013). Phosphorylation-regulated binding of RNA polymerase II to fibrous polymers of low-complexity domains. Cell 155, 1049–1060. 24267890 10.1016/j.cell.2013.10.033 PMC4010232

[mjab023-B15] Lin C. , Garrett A.S., De Kumar B., et al (2011). Dynamic transcriptional events in embryonic stem cells mediated by the super elongation complex (SEC). Genes Dev. 25, 1486–1498. 21764852 10.1101/gad.2059211 PMC3143939

[mjab023-B16] Lin C. , Garruss A.S., Luo Z., et al (2013). The RNA Pol II elongation factor Ell3 marks enhancers in ES cells and primes future gene activation. Cell 152, 144–156. 23273992 10.1016/j.cell.2012.12.015 PMC3556173

[mjab023-B17] Lin C. , Smith E.R., Takahashi H., et al (2010). AFF4, a component of the ELL/P-TEFb elongation complex and a shared subunit of MLL chimeras, can link transcription elongation to leukemia. Mol. Cell 37, 429–437. 20159561 10.1016/j.molcel.2010.01.026 PMC2872029

[mjab023-B18] Lu H. , Yu D., Hansen A.S., et al (2018). Phase-separation mechanism for C-terminal hyperphosphorylation of RNA polymerase II. Nature 558, 318–323. 29849146 10.1038/s41586-018-0174-3 PMC6475116

[mjab023-B19] Luo Z. , Lin C., Guest E., et al (2012a). The super elongation complex family of RNA polymerase II elongation factors: gene target specificity and transcriptional output. Mol. Cell. Biol. 32, 2608–2617. 22547686 10.1128/MCB.00182-12 PMC3434493

[mjab023-B20] Luo Z. , Lin C., Shilatifard A. (2012b). The super elongation complex (SEC) family in transcriptional control. Nat. Rev. Mol. Cell Biol. 13, 543–547. 22895430 10.1038/nrm3417

[mjab023-B21] McSwiggen D.T. , Mir M., Darzacq X., et al (2019). Evaluating phase separation in live cells: diagnosis, caveats, and functional consequences. Genes Dev. 33, 1619–1634. 31594803 10.1101/gad.331520.119 PMC6942051

[mjab023-B22] Nguyen V.T. , Kiss T., Michels A.A., et al (2001). 7SK small nuclear RNA binds to and inhibits the activity of CDK9/cyclin T complexes. Nature 414, 322–325. 11713533 10.1038/35104581

[mjab023-B23] Ni Z. , Saunders A., Fuda N.J., et al (2008). P-TEFb is critical for the maturation of RNA polymerase II into productive elongation in vivo. Mol. Cell. Biol. 28, 1161–1170. 18070927 10.1128/MCB.01859-07 PMC2223398

[mjab023-B24] Plet A. , Eick D., Blanchard J.M. (1995). Elongation and premature termination of transcripts initiated from c-fos and c-myc promoters show dissimilar patterns. Oncogene 10, 319–328. 7838531

[mjab023-B25] Sabari B.R. , Dall'Agnese A., Boija A., et al (2018). Coactivator condensation at super-enhancers links phase separation and gene control. Science 361, eaar3958. 29930091 10.1126/science.aar3958 PMC6092193

[mjab023-B26] Shin Y. , Brangwynne C.P. (2017). Liquid phase condensation in cell physiology and disease. Science 357, eaaf4382. 28935776 10.1126/science.aaf4382

[mjab023-B27] Steurer B. , Janssens R.C., Geverts B., et al (2018). Live-cell analysis of endogenous GFP-RPB1 uncovers rapid turnover of initiating and promoter-paused RNA Polymerase II. Proc. Natl Acad. Sci. USA 115, E4368–E4376. 29632207 10.1073/pnas.1717920115 PMC5948963

[mjab023-B28] Vos S.M. , Farnung L., Urlaub H., et al (2018). Structure of paused transcription complex Pol II‒DSIF‒NELF. Nature 560, 601–606. 30135580 10.1038/s41586-018-0442-2 PMC6245578

[mjab023-B29] Wada T. , Takagi T., Yamaguchi Y., et al (1998). DSIF, a novel transcription elongation factor that regulates RNA polymerase II processivity, is composed of human Spt4 and Spt5 homologs. Genes Dev. 12, 343–356. 9450929 10.1101/gad.12.3.343 PMC316480

[mjab023-B30] Wan L. , Chong S., Xuan F., et al (2020). Impaired cell fate through gain-of-function mutations in a chromatin reader. Nature 577, 121–126. 31853060 10.1038/s41586-019-1842-7 PMC7061414

[mjab023-B31] Yamaguchi Y. , Takagi T., Wada T., et al (1999). NELF, a multisubunit complex containing RD, cooperates with DSIF to repress RNA polymerase II elongation. Cell 97, 41–51. 10199401 10.1016/s0092-8674(00)80713-8

[mjab023-B32] Yang Z. , Yik J.H., Chen R., et al (2005). Recruitment of P-TEFb for stimulation of transcriptional elongation by the bromodomain protein Brd4. Mol. Cell 19, 535–545. 16109377 10.1016/j.molcel.2005.06.029

[mjab023-B33] Yang Z. , Zhu Q., Luo K., et al (2001). The 7SK small nuclear RNA inhibits the CDK9/cyclin T1 kinase to control transcription. Nature 414, 317–322. 11713532 10.1038/35104575

